# The Association Between Matrix Metalloproteinase-1, -2, -3, -9, and -12 Gene Polymorphisms and Atrial Fibrillation

**DOI:** 10.3390/ijms27156710

**Published:** 2026-07-27

**Authors:** Robert Błaszczyk, Sebastian Sawonik, Izabela Korona-Głowniak, Anna Wysocka, Monika Czuba, Małgorzata Świstowska, Olgierd Król, Janusz Kocki, Andrzej Wysokiński, Andrzej Głowniak

**Affiliations:** 1 Department of Electrocardiology, Medical University of Lublin, University Centre of Cardiology and Cardiac Surgery, 20-059 Lublin, Poland; robert.blaszczyk@umlub.edu.pl (R.B.); anna.wysocka@umlub.pl (A.W.); andrzej.glowniak@umlub.edu.pl (A.G.); 2 Department of Cardiology, Medical University of Lublin, University Centre of Cardiology and Cardiac Surgery, 20-093 Lublin, Poland; olgierdkrol96@gmail.com; 3 Department of Pharmaceutical Microbiology, Faculty of Pharmacy, Medical University of Lublin, 20-093 Lublin, Poland; izabela.korona-glowniak@umlub.edu.pl; 4 Department of Internal Medicine and Internal Nursing, Faculty of Health Sciences, Medical University of Lublin, 20-059 Lublin, Poland; 5 Department of Clinical Genetics, Medical University of Lublin, 20-080 Lublin, Poland; monika.czuba@umlub.edu.pl (M.C.); malgorzata.swistowska@umlub.edu.pl (M.Ś.); janusz.kocki@umlub.pl (J.K.)

**Keywords:** atrial fibrillation, matrix metalloproteinases, polymorphism, extracellular matrix, fibrosis, gene–gene interaction, haplotype

## Abstract

Atrial fibrillation (AF) is a prevalent cardiac arrhythmia associated with significant morbidity and mortality. Structural remodeling of the left atrium, particularly myocardial fibrosis, plays a key role in AF pathogenesis. Matrix metalloproteinases (MMPs) are critical regulators of extracellular matrix remodeling and may contribute to atrial fibrosis through genetic variation. This case–control study included 179 patients with AF and 56 controls. Eight polymorphisms across five MMP genes (MMP1, MMP2, MMP3, MMP9, and MMP12) were analyzed using PCR-based methods. Associations between single-nucleotide polymorphisms (SNPs), AF susceptibility, recurrence, haplotypes, and gene–gene interactions were assessed. The study population was ethnically homogeneous (Polish), minimizing population stratification bias. No significant differences in allele frequencies were observed between AF and control groups in univariate analysis. However, multivariable logistic regression revealed significant associations for MMP1 rs1799750 and MMP2 rs243864 under recessive inheritance models. Haplotype analysis demonstrated a significant global association with AF (*p* = 0.027), with specific haplotypes showing markedly increased risk. Multifactor dimensionality reduction identified significant gene–gene interactions, particularly involving SNPs in MMP1, MMP2, MMP3, and MMP12. These findings support a polygenic model of AF susceptibility involving extracellular matrix remodeling pathways and highlight the importance of multi-locus genetic analyses.

## 1. Introduction

Atrial fibrillation represents a major global healthcare burden in many countries. Over 30 million patients worldwide suffer from atrial fibrillation. Forecasts indicate that by 2060, there will be between 14 and 18 million cases of this type of heart rhythm disorder in Europe alone, and in the US alone there will be between 6 and 12 million patients by 2050 [1]. Improved healthcare systems in developed countries contribute to higher detection rates of atrial fibrillation. Early detection of arrhythmia is important because AF is associated with a 5-fold increase in the risk of stroke. And 20% of all strokes in the UK occur in patients with AF [2]. The percentage of patients with asymptomatic AF ranges from 11.5% to as high as 40%. Short episodes of AF lasting several minutes (5–6 min) increase the risk of stroke by approximately 2.5 times [3]. The frequent occurrence of arrhythmia, as well as thromboembolic complications in untreated AF, prompts an active search for methods of screening for clinically silent AF. Left atrial (LA) remodeling is a risk factor for atrial fibrillation (AF) and is indirectly associated with the occurrence of ischemic stroke (IS). Left atrial remodeling represents the link between morphological changes and electrophysiological abnormalities that contribute to the onset and maintenance of atrial fibrillation, creating a vicious cycle between atrial cardiomyopathy (AC) and cardiac arrhythmias [4]. Atrial myocardial fibrosis leads to a slowing of impulse conduction, which has a proarrhythmic effect and increases the risk of arrhythmias. Newman et al. confirm this hypothesis, stating that LA scarring and fibrosis leads to a shortened atrial refractory period, slowed conduction velocity and, consequently, atrial fibrillation [5]. Therefore, the present study aimed to investigate the genetic basis of increased myocardial fibrosis. But do the benefits of active screening for left atrial remodeling and detection of asymptomatic arrhythmia become standard practice, or do the costs of such an undertaking significantly outweigh the potential benefits? Lowres et al., in a meta-analysis based on 30 available scientific studies based on pulse palpation or ECG, indicate the detection of previously undiagnosed AF in approximately 1% of patients, and in the group over 65 years of age, in 1.4% [6,7]. Fitzmaurice et al., in a screening study involving 14,802 patients in 50 primary care practices, indicate a detection rate of atrial fibrillation in 1.63% of previously undiagnosed individuals [8]. A natural response to this social need is to search for genetic differences that allow the identification of individuals at increased risk of developing atrial fibrillation in the future. Polymorphisms in MMP genes contribute to ischemic stroke (IS) being the most serious AF complication and additionally are associated with disorders that are risk factors of IS in patients with AF such as diabetes or heart failure. Matrix metalloproteinases (MMPs) are zinc-dependent endopeptidases responsible for the degradation and remodeling of the extracellular matrix (ECM) in cardiac tissue. Elevated MMP-9 levels occur in specific risk groups, such as obese individuals who have a profile of early atherosclerosis development. Furthermore, it has been shown that serum MMP-9 levels were elevated in patients with pronounced carotid atherosclerosis compared to healthy patients and that only MMP-9 levels were elevated in contrast to other proposed biomarkers such as MMP-1 and MMP-3 [9]. A study by Nilsson et al. involving 1500 patients and measuring plasma levels of MMP-1, -3, -7, -10, and -12 showed that MMP-7 and MMP-12 were elevated in patients with type 2 diabetes, which is associated with atherosclerosis and coronary events [10]. In atrial fibrillation and related pathologic states, dysregulated MMP activity—particularly increased MMP-2 and MMP-9 expression with reduced TIMP inhibition—leads to excessive ECM turnover and structural remodeling. This imbalance promotes atrial fibrosis by triggering fibroblast activation and collagen deposition, ultimately contributing to electrical and mechanical dysfunction of the atria [11]. Fan et al. [12] observed that greater susceptibility to stroke was also detected in polymorphic variants of several MMP genes. The evaluation of SNPs in four genes (rs1799750 in MMP-1, rs2285053, rs243865, rs2241145 in MMP-2, rs17576 in MMP-9, rs2276109, rs660599 and rs652438 in MMP-12) and their interactions allowed detection of an association between the rs17576 AG and GG genotypes and an increased risk of ischaemic stroke (IS), as well as a significant interaction between MMP-9 rs17576 and MMP-12 rs660599, also correlating with a higher risk of IS [12]. Gajewska et al. indicate that another SNP associated with the occurrence of TDM2 in humans is the MMP-2-1575-G/A (rs243866) polymorphism and two MMP-9 polymorphisms: −1562 C/T (rs3918242) and +279 A/G (rs17576) [13]. MMP-3 was included because it degrades multiple extracellular matrix components and can activate other pro-MMPs, thereby amplifying proteolytic cascades involved in structural tissue remodeling. Because atrial fibrosis and extracellular matrix dysregulation are central components of the atrial fibrillation substrate, MMP-3 represents a biologically plausible candidate even though direct AF-specific genetic evidence remains limited. MMP-12 was selected as an inflammation-related metalloproteinase with elastolytic and extracellular matrix-remodeling properties. Although direct evidence linking MMP-12 specifically to atrial fibrillation is still limited, its established role in cardiovascular matrix remodeling supports its inclusion in a candidate-gene panel focused on extracellular matrix turnover in AF [14,15,16]. On the other hand, Zhao et al. indicate that polymorphism MMP3 (rs522616) was associated with a significantly decreased risk of brain arteriovenous malformation [17]. Beber et al. do not exclude the possibility that −1575G > A (rs243866), −1059G > A (rs17859821), and −790G > T (rs243864) polymorphisms in the MMP-2 gene might be associated with HF prognosis in Caucasian-Brazilians with reduced LVEF [18]. To summarize, the selected SNPs were chosen because they are extensively studied functional polymorphisms of matrix metalloproteinase (MMP) genes and they are known or suspected to influence MMP transcription, expression, and/or enzymatic activity. Since MMPs play an important role in extracellular matrix turnover, inflammation, fibrogenesis, and tissue repair processes, genetic variants affecting their expression may contribute to interindividual differences in circulating MMP levels and atrial fibrillation occurrence and recurrence highly dependent on atrial remodeling. Further details are available in Table S1. GRCh38 coordinates for all rsIDs, enabling the unambiguous identification of variants and serving as the basis for the interpretation of local LD and haplotype analyses within the cluster, are presented in the Supplementary Materials (Tables S2 and S3).

Therefore, this study aimed to evaluate the association between selected MMP gene polymorphisms (as a marker of increased myocardial fibrosis) and AF risk in a Polish cohort, and to explore haplotype structure and gene–gene interactions to better understand the complex genetic architecture underlying atrial remodeling.

## 2. Results

A total of 179 patients with atrial fibrillation (AF) and 56 controls were included. The AF cohort was significantly older than controls (65.6 ± 11.5 vs. 49.6 ± 19.4 years, *p* < 0.001) and had a higher BMI (27.9 ± 3.8 vs. 24.7 ± 2.9 kg/m^2^, *p* = 0.007). Sex distribution did not differ between groups. Among AF patients, the median time from diagnosis was 4.0 years (IQR 3.0–6.0), with paroxysmal AF accounting for 73.2% of cases. Comorbidities were common: chronic kidney disease (35.2%), coronary artery disease (32.4%), previous stroke (8.9%), transient ischemic attack (29.1%), and heart failure (78.8%). Echocardiographic assessment showed preserved median LVEF (60%) and enlarged left atrial dimensions (mean LA volume 118.8 ± 27.4 mL; LAVI 60.9 ± 14.7 mL/m^2^). The data is presented comprehensively in Table 1 and Table 2.

All investigated polymorphisms (SNP1–SNP8) were in Hardy–Weinberg equilibrium in both AF and control groups. No statistically significant differences in genotype or allele frequencies were observed between AF patients and controls for any of the investigated polymorphisms in the univariate analyses. Numerical differences were observed for several variants; however, these did not reach statistical significance and should be interpreted as exploratory observations. For MMP1 rs1799750 (SNP1), a non-significant numerical difference toward more frequent carriage of the 2G allele in AF patients was observed (54.6% vs. 45.5%, *p* = 0.10). Similar non-significant trends toward a higher prevalence of risk alleles in AF patients were noted for SNPs in MMP2 (rs243866, rs17859821, rs243864), MMP3 (rs522616), MMP9 (rs378768, rs17576), and MMP12 (rs2276109). Information is contained in Table 3 and Table 4. Further information on the study of the association between the SNPs examined in this study and atrial fibrillation can be found in Tables S4–S10 in the Supplementary Materials.

Due to the inability to clearly determine AF burden (due to the need for ILR implantation), an attempt was made to quantify the severity of atrial fibrillation by dividing patients into groups with AF recurrence within the last 3, 6, and 12 months and those without AF recurrence during that time (data collected from medical interviews regarding the presence of documented arrhythmia at 3, 6 and 12 months as documented by a Holter monitor, a 12-lead ECG or ECG monitoring). Among the risk alleles studied, there was no statistically significant difference in the occurrence of the listed genes between the two subgroups (Table 5). However, a slightly higher frequency of the risk allele distribution for MMP2—rs243866r was observed in patients with AF recurrence after 6 and 12 months (genotypes G/A and A/A), and for MMP2—s17859821 (genotype G/A and A/A) in patients with AF recurrence after 3 and 6 months. Genotypes T/C of MMP3—rs522616 and MMP12—rs2276109 T/C were more frequent in patients who experienced AF recurrence within 3, 6, and 12 months of observation. MMP9—rs17576 A/G was more frequent in patients with AF recurrence after 6 and 12 months (Table 5).

Propensity score matching was performed to reduce imbalance between the AF and control groups. Matching with replacement altered the composition of the control sample (control counts/weights increased), resulting in notable changes in genotype distributions for several SNPs (Tables S14 and S15). For some SNPs (e.g., SNP2, SNP4, SNP7, SNP5), the SMDs became large (>0.10, in several cases ≈0.23–0.39) after matching, indicating poor balance for those genotypes in the matched sample. A few SNPs (SNP6, SNP8) show an acceptable balance after matching. These results indicate that the matching procedure substantially altered the control profile for multiple SNPs and did not uniformly improve covariate/genotype balance. In our data, the control group from the original (unmatched) sample appears to be better balanced with the AF group than the propensity-score-matched sample. This conclusion is based on a comparison of standardized mean differences (SMDs) and genotype distributions before and after matching: several covariates/SNPs that had small SMDs in the original cohort show substantially larger SMDs after matching, indicating worse balance post-PSM.

Logistic regression with AF as the dependent variable was shown for the unadjusted cohort and for the propensity-score-matched cohort. Matching changed effect estimates substantially for several SNPs (notably SNP1, SNP5, SNP7, SNP8, and SNP4), increasing significance and effect sizes in the matched samples (Table S15). Several ORs in the matched model have very large magnitudes and wide CIs, suggesting possible instability due to sparse cells and influential observations.

The association between the MMP polymorphisms and atrial fibrillation (AF) was assessed under five inheritance models (Tables S4–S13). Following re-evaluation of genotype coding and inheritance model specification, no consistent evidence was found to support a protective effect of the 2G allele. However, in the codominant model, genotype 2G/2G vs. 1G/1G shows a higher, but not significant, risk (OR = 0.30, 95% CI 0.10–0.94, *p* = 0.078); the homozygote contrast is significant. In the dominant model, carriers of 1 or more copies of 2G (1G/2G + 2G/2G) have increased AF risk versus 1G/1G (OR = 0.39, 95% CI 0.17–0.92, *p* = 0.032). A significant log-additive model (OR = 0.54, 95% CI 0.30–0.95, *p* = 0.028) indicated a dose-dependent protective effect per 2G allele.

Moreover, for MMP2—rs243864, the recessive model showed a significant association of homozygous G/G with decreased AF risk (6.2% vs. 10.7% in controls; OR = 5.30, 95% CI 1.13–24.81, *p* = 0.035). In the codominant model, the global test was not significant (*p* = 0.085), but the G/G versus T/T comparison yielded OR = 5.88 (95% CI 1.22–28.39).

**Figure 1:**
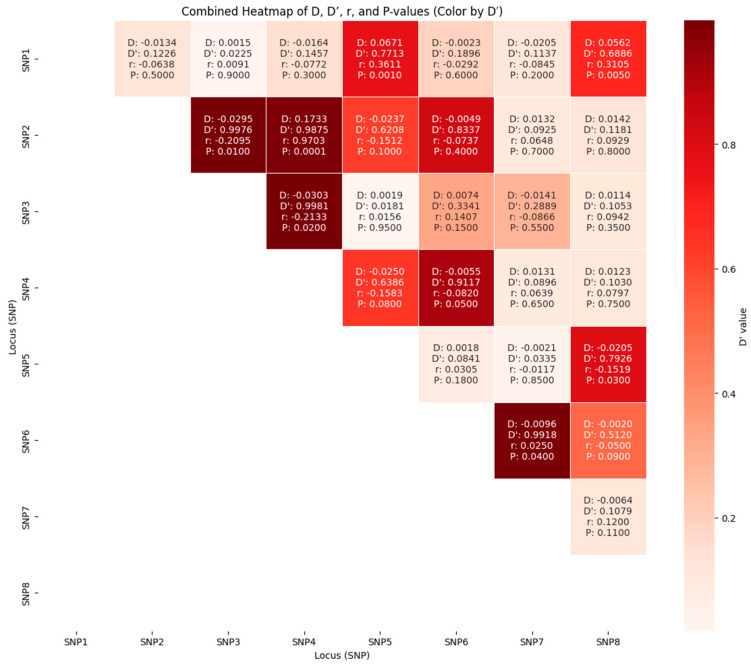
Triangular linkage disequilibrium heatmap.

Pairwise linkage disequilibrium (LD) statistics (D, D’, r) were calculated for eight SNPs. D’ statistic values indicated several regions of moderate to strong LD: SNP1–SNP5 (D’ = 0.7713) and SNP1–SNP8 (D’ = 0.6886) showed strong LD. SNP5–SNP8 (D’ = 0.7926) also displayed high LD. Additional moderate associations were observed between SNP2–SNP5 (D’ = 0.6208), SNP2–SNP6 (D’ = 0.8337), SNP4–SNP5 (D’ = 0.6386), and SNP6–SNP7 (D’ = 0.9918). The r statistic supported these patterns, with the strongest correlations found for SNP1–SNP5 (r = 0.3611) and SNP1–SNP8 (r = 0.3105), indicating that these loci are inherited together more often than expected by chance. Together, these results suggest the presence of at least one haplotype block containing SNP1, SNP5, and SNP8, and possibly extending to SNP2 and SNP6, given the moderate D’ and significant associations. The clustering of significant LD in these regions implies physical proximity or selective co-inheritance, which may be relevant to downstream association analyses (Figure 1).

Haplotype association analysis was conducted to examine the relationship between haplotypes across SNP1–SNP8 and AF occurrence in 235 patients, adjusting for sex and age. The global haplotype association was statistically significant (*p* = 0.027), indicating that variation in this haplotype block may influence the occurrence of AF (Table S8). The reference haplotype H1: 2G–G–G–T–T–G–G–T, with a frequency of 16.6%, was used as the baseline for comparison. The haplotype H12 (1G-G-G-T-T-G-G-T; frequency 2.06%) and H16 (1G-A-G-G-T-G-A-C; frequency 1.4) revealed significantly increased odds of AF occurrence (*p* < 0.0001). Several haplotypes showed notably decreased odds of AF occurrence: H4 (1G–G–G–T–T–G–A–T; frequency = 8.6%) was associated with a 0.12-fold higher odds of AF (95% CI: 0.02–0.59, *p* = 0.0095). H7 (1G–A–G–G–T–G–G–T; frequency = 4.5%) showed association with AF, with a 0.03-fold increase in odds (95% CI: 0.00–0.29, *p* = 0.0031). Overall, these findings suggest that specific multi-locus combinations—particularly H12 and H16—may significantly influence AF occurrence, warranting further investigation in larger cohorts.

Multifactor dimensionality reduction (MDR) analysis was performed to identify SNP-SNP interactions that affect the risk of atrial fibrillation (Table S9). MDR analysis identified statistically significant gene–gene interactions (epistasis) between SNP1 + SNP8 (CVC 7/10; OR = 3.6, 95% CI 1.1–12.3) and SNP1 + SNP4 + SNP5 (CVC 3/10; OR = 2.5, 95% CI 1.1–5.5). However, moderate test accuracy values (all below 60%) suggest that these gene combinations explain only part of the variance in disease risk, and environmental and clinical factors or other genes also play a significant role.

## 3. Discussion

The risk of atrial fibrillation recurrence is associated with variants of metalloproteinase genes. Lombardi et al. [19] in a prospective study, assessed the relationship between MMP-1 and MMP-3 polymorphisms in patients with persistent atrial fibrillation whose sinus rhythm was restored by electrical cardioversion (ECV). The authors observed an increased risk of atrial fibrillation recurrence in carriers of both the 5A and 1G alleles compared to people without these alleles [19,20]. Huxley et al. report that elevated activity of MMP-9 was associated with increased risk of AF [21].

This study provides novel evidence supporting the contribution of matrix metalloproteinase (MMP) gene polymorphisms to atrial fibrillation (AF) susceptibility in a cohort recruited from Polish population. We evaluated eight polymorphisms across five metalloproteinase genes (MMP1, MMP2, MMP3, MMP9, and MMP12) and their potential associations with atrial fibrillation (AF), AF recurrence, and gene–gene interactions. Although no significant differences in allele frequencies were observed between AF patients and controls in the initial allelic analysis, deeper genetic modeling revealed specific variants and multi-locus structures associated with AF susceptibility. The most robust single-locus associations were observed for MMP1 rs1799750 and MMP2 rs243864, both demonstrating significance under the recessive inheritance model. The 1G/1G genotype in MMP1 and the G/G genotype in MMP2 were associated with higher odds of AF, suggesting that reduced promoter functionality or altered transcriptional activity—previously attributed to these variants in experimental studies—may contribute to atrial remodeling. Although individual SNPs in other metalloproteinases (MMP3, MMP9, MMP12) did not reach statistical significance, the observed numerical enrichment of risk alleles in AF patients suggests that subtle or cumulative effects may exist but remain underpowered in single-variant analyses. Similarly, Buckley et al. reported that higher MMP2 levels are associated with heart failure with preserved ejection fraction and left atrial dysfunction, as well as AF (the highest MMP-2 quartile was associated with greater risk of incident AF (1.44 [95% CI, 1.18–1.77]) [22]. Diao et al. report that, compared to the sinus rhythm group, protein and mRNA expression levels of MMP-2 were evidently increased in the 3 AF groups, concluding that elevated MMP2 levels are associated with the onset of atrial fibrillation [23]. Hsiao and colleagues investigated the association between MMP9 and the occurrence of arrhythmia. The expression of MMP9 was higher in fibrillating atrial tissue than in sinus rhythm. However, there was no significant difference in the distribution of rs3918242 genotypes and allele frequencies between the control group and the AF group [24].

Importantly, haplotype analysis identified significant multi-allelic patterns, indicating that combinations of MMP polymorphisms may better capture the genetic architecture predisposing to AF. Haplotypes H4 and H7 were strongly associated with AF, with odds ratios exceeding those of any single SNP, underscoring the relevance of multi-locus effects. These findings are consistent with the notion that extracellular matrix (ECM) dynamics are governed not by single enzymatic pathways but by coordinated interactions among MMP family members. On the other hand, the haplotype analysis should be interpreted cautiously. Given the relatively modest sample size and the number of possible multi-locus combinations, there is a risk of overfitting, particularly for low-frequency haplotypes. Several haplotypes identified as significant were rare (<2–3%), which may have resulted in unstable effect estimates and limited reproducibility. Therefore, these associations should be considered exploratory and hypothesis-generating rather than confirmatory, and they require validation in larger, independent cohorts.

The analysis of linkage disequilibrium further supports this interpretation, revealing moderate to strong LD across SNP pairs—most notably involving SNP1, SNP5, SNP8, and several MMP2-linked variants. This suggests the existence of functional haplotype blocks rather than isolated allelic events. The presence of clustered LD regions may reflect shared transcriptional regulation, proximity within regulatory domains, or coordinated evolutionary pressures. The search for risk alleles and specific haplotypes that increase the risk of cardiovascular disease is a well-known approach in the literature. Purkait et al. identified four SNPs (rs11568020: A-152G and rs5050: A-20C in the promoter; rs4762 and rs699 in exon 2) and three haplotypes (H4, H7, and H8) that showed a stronger positive association with hypertension. In contrast, haplotype H2 showed a protective effect against hypertension [25].

The multifactor dimensionality reduction (MDR) models provided additional evidence for genetic interactions influencing AF risk. Models incorporating combinations of SNP1, SNP3, SNP5, and SNP8 yielded statistically significant associations and improved predictive metrics compared with single-SNP analyses. Notably, gene–gene interactions were strongest in the 6-month recurrence subgroup, where the SNP3 + SNP5 and SNP1 + SNP3 + SNP5 models achieved the highest cross-validation consistency. These results suggest that epistatic effects may contribute meaningfully to the risk of arrhythmia recurrence, even when individual SNPs do not independently predict outcomes [20]. In the presented paper, MDR findings are exploratory and hypothesis-generating. Confirmation in larger, independent datasets is necessary before drawing firm conclusions about their biological or clinical significance.

Despite the significant findings in some genetic models, the overall predictive accuracy of MDR models remained modest, indicating that genetic predisposition related to metalloproteinases captures only part of the variability in AF risk and recurrence. This is consistent with the multifactorial pathophysiology of AF, where structural remodeling, electrophysiological alterations, comorbidities, inflammation, and environmental influences interact to determine arrhythmogenic susceptibility. In the present study, AF patients demonstrated a high prevalence of comorbid conditions known to promote atrial remodeling, particularly heart failure, chronic kidney disease, and metabolic abnormalities. These clinical factors may overshadow isolated genetic effects or interact with them in ways not detectable by current modeling.

No genotype or allele differences reached significance between patients with and without AF recurrence at 3, 6, or 12 months. However, recurrence groups consistently demonstrated numerically higher frequencies of several risk alleles, which—although not statistically conclusive—align with the MDR findings suggesting the presence of interaction-driven rather than single-variant recurrence risk.

Taken together, the results indicate that while individual MMP polymorphisms show limited association with AF, specific variants in MMP1 and MMP2, as well as multi-locus genetic configurations, may contribute to susceptibility. The study also highlights the importance of moving beyond traditional single-SNP analyses to incorporate haplotype structure and epistatic interactions, which may uncover mechanistic pathways that are not apparent when loci are examined in isolation.

Functional consequences of these polymorphisms were not assessed and require further investigation. Future studies with larger sample sizes and functional correlates are warranted to clarify the biological relevance of the identified haplotypes and interaction models, and to determine whether these findings can be integrated into clinical risk stratification or precision-medicine approaches to AF. Furthermore, the occurrence of atrial fibrillation is influenced by numerous factors (age, race, hypertension, left atrial size, and others). Myocardial fibrosis not only contributes to the development of arrhythmias but also increases the risk of other cardiovascular diseases. There is a need for extensive research into the relationship between these factors, the occurrence of arrhythmias, and the presence of specific metalloproteinase genes.

## 4. Materials and Methods

### 4.1. Study Population

This case–control study included 179 patients with atrial fibrillation (AF) and 56 control subjects recruited from the Department of Cardiology, Medical University in Poland, between 2023 and 2025. AF diagnosis was based on 12-lead ECG documentation. The control group consisted of individuals with no history of AF or other supraventricular arrhythmias. Exclusion criteria for both groups included: congenital heart disease, cardiomyopathy, significant valvular defects, acute myocardial infarction within the last 6 months, inflammatory or autoimmune diseases, malignancies, and chronic liver or renal failure. Any recurrences of atrial fibrillation at 3, 6 and 12 months were investigated using Holter ECG, a 12-lead ECG or ECG monitoring.

Demographic and clinical data (age, sex, BMI, hypertension, diabetes, coronary artery disease, and echocardiographic parameters) were collected using standardized questionnaires and medical records. All participants provided written informed consent prior to enrollment. Patients were surveyed to determine whether they had experienced any recurrence of atrial fibrillation three months and six months after the start of the study. The study protocol was approved by the Local Bioethics Committee of the Medical University of Lublin (approval no. KE-0254/219/10/2023).

Peripheral venous blood samples (5 mL) were collected in EDTA tubes. DNA was isolated from 500 µL of whole blood using the Blood Mini kit (A&A Biotechnology, Gdańsk, Poland) (column-based method) followed by PCR reaction using commercial probes for selected DNA polymorphisms in a StepOne plus device (Applied Biosystems, Foster City, CA, USA). Polymorphic sites of metalloproteinase (MMP) promoter regions were identified: MMP-1: −1607 1G/2G, MMP-2: −1575 G/A (rs243866); −1059 G/A (rs17859821), −790 G/T (rs243864); MMP-3: −1171 (5A/6A); MMP-9: −1562 C/T (rs3918242); rs378768 G/A; rs1056628 A/C; MMP-12: −82 A/G.

### 4.2. Genotyping

Genotyping was performed by real-time PCR using TaqMan allelic discrimination probes (Applied Biosystems, Foster City, CA, USA), depending on the locus. For quality control, approximately 10% of samples were randomly re-genotyped, yielding 100% concordance. Investigators conducting the genotyping analyses had no access to clinical information during the experimental procedures. Further details regarding DNA isolation are available in the Supplementary Materials (Table S13).

### 4.3. Statistical Analysis

Statistical analysis was performed using Spotfire Statistica 14.4 (New York, NY, USA) and SNPStats 1.62.0. www.snpstats.net (accessed on 10 August 2025). Unless otherwise indicated, data are given as mean ± SD or median (IQR). Categorical variables are expressed as frequencies and percentages. Genotype distributions were compared between the AF and control groups using the chi-square test or Fisher’s exact test, as appropriate. In addition, genotype distributions were compared between patients with and without AF recurrence at 3, 6, and 12 months after the initial diagnosis.

Propensity-score matching was performed to reduce the frequency imbalance between the AF and control group that may be influenced by confounders. Logistic regression models, with the AF group as dependent variable, were used to estimate propensity scores. The variables identified as potential confounders were entered in the propensity score model. The covariates were: age, sex, BMI, and MMP alleles. Using the estimated propensity score, patients from the control group were matched to AF patients via nearest-neighbor matching at a 1:1 ratio with replacement. Balance between the matched groups was assessed using standardized mean differences (SMDs) for variables included in the propensity score model. A variable was considered to be well-balanced between the groups if the SMD < 0.10. The distributions of the propensity scores in both exposure groups were visualized to evaluate whether the positivity assumption was violated.

SNP–AF associations were evaluated under five inheritance models (codominant, dominant, recessive, overdominant, and log-additive) using multivariable logistic regression with AF status as the dependent variable and age and sex as covariates. Fisher’s exact test for Hardy–Weinberg equilibrium (HWE) for a single-nucleotide polymorphism in the study group was evaluated (assessed separately in AF cases and controls). All investigated polymorphisms fulfilled HWE expectations in both groups (*p* > 0.05). HWE is commonly used for quality control of genotyping: it indicates inbreeding, population stratification, and systematic genotyping errors in unrelated individuals. To determine the association between MMP-1, MMP-2, MMP-3, MMP-9 and MMP-12 genotypes and the risk of AF, odds ratios (ORs) with corresponding 95% confidence intervals (CIs) were calculated. Statistical significance was defined as any *p*-value less than 0.05.

Because this was an exploratory candidate-gene association study, no formal adjustment for multiple testing was performed. Therefore, all reported *p*-values should be interpreted as exploratory and hypothesis-generating.

### 4.4. Limitations

Several limitations should be acknowledged. The marked age difference between AF patients and controls represents an important limitation. Although all regression analyses were adjusted for age and sex, residual confounding cannot be completely excluded. Similarly, the relatively small control cohort and the low frequency of several homozygous variant genotypes may have reduced statistical power and increased the risk of unstable effect estimates. Differences between study groups, including age and comorbidities, were adjusted for in multivariable models but may still influence the results. The study sample size, though sufficient for detecting moderate effects, limits the power for rare allele analysis. Larger multicenter studies are needed to confirm these findings. Furthermore, only selected MMP polymorphisms were analyzed; additional variants or regulatory SNPs (e.g., in promoter or enhancer regions) may also influence gene expression. Additionally, plasma MMP and TIMP levels were not measured, precluding direct correlation between genotype and enzyme activity. Moreover, the cross-sectional design prevents causal inference. Longitudinal studies are required to assess whether these polymorphisms predict incident or recurrent AF.

An additional limitation is that no correction for multiple comparisons was applied despite the large number of statistical tests performed. Consequently, some statistically significant associations may represent false-positive findings and should be interpreted with caution. Replication in larger, independent cohorts with appropriate adjustment for multiple testing is required to confirm the present findings.

Despite these limitations, the study provides valuable insights into the genetic determinants of ECM remodeling in AF and represents one of the first comprehensive analyses of MMP polymorphisms in a Central European cohort. Despite differences between the study group and the control group, the study provides new insight into the genetic differences between people with atrial fibrillation and those without this diagnosis. Other conditions, such as heart failure and hypertension, which do not occur in the control group, have an impact on the occurrence of atrial fibrillation. However, they do not affect the genetic differences between the groups.

## 5. Conclusions

In this study, we comprehensively evaluated the role of metalloproteinase gene polymorphisms in atrial fibrillation. While the overall allele frequencies of the investigated variants did not differ significantly between AF patients and controls, specific genotypes in MMP1 (rs1799750) and MMP2 (rs243864) demonstrated significant associations with AF in recessive genetic models. These findings suggest that select MMP polymorphisms may contribute to AF susceptibility through their influence on extracellular matrix remodeling.

Haplotype analysis further revealed that multi-locus combinations across MMP genes exert a stronger association with AF than individual SNPs, identifying high-risk haplotypes with markedly elevated odds ratios. In addition, multifactor dimensionality reduction analysis indicated significant gene–gene interactions affecting AF risk and recurrence, particularly involving SNPs in MMP1, MMP2, MMP3, and MMP12.

Although no single SNP predicted short- or long-term recurrence, the consistent numerical enrichment of risk alleles and the presence of significant interaction models suggest that AF recurrence may be influenced by polygenic effects rather than isolated genetic variants.

Overall, these results indicate that the genetic architecture underlying MMP-related extracellular matrix regulation may play a contributory role in AF development. Multi-locus analyses, including haplotypes and epistatic models, appear more informative than individual polymorphisms alone and may offer additional insight into AF susceptibility pathways. Further studies in larger and independent cohorts are needed to validate these associations and clarify their potential clinical relevance.

## Figures and Tables

**Table 1:** Baseline sociodemographic characteristics of the studied and control group.

Parameter		AF Patients (*n* = 179)		Controls (*n* = 56)		*p* Value
		Mean ± SD	Median (IQR)	Mean ± SD	Median (IQR)	
Age (years)		65.6 ± 11.5	68.0 (59.0–74.0)	49.6 ± 19.4	44.0 (33.0–69.0)	<0.001
Age groups	(23–55)	32 (18%)		37 (66%)		
	(55–66)	45 (25%)		5 (5%)		
	(66–73)	50 (28%)		6 (11%)		
	(73–90)	52 (29%)		10 (18%)		
Male Sex (%)		115 (64.25)		12 (21.4)		0.61
BMI (kg/m^2^)		27.9 ± 3.8	27.8 (24.9–30.5)	24.7 ± 2.9	23.7 (22.7–26.6)	0.007

Abbreviations: AF—atrial fibrillation; IQR—interquartile range; SD—standard deviation. The *p*-value was calculated using the Chi-square test.

**Table 2:** Baseline characteristics of the studied AF group.

Parameter	AF Patients (*n* = 179)	
	Mean ± SD	Median (IQR)
Time from AF diagnosis (years)	4.6 ± 2.5	4.0 (3.0–6.0)
**Biochemical parameters**		
WBC (109/L)	6.6 ± 2.1	6.2 (5.0–7.3)
HGB (g/dL)	14.1 ± 1.6	14.1 (13.2–15.1)
HCT (%)	42.1 ± 4.4	41.9 (39.4–44.8)
RDW (%)	13.8 ± 1.3	13.6 (13.0–14.2)
PLT (109/L)	220.1 ± 60.8	218.0 (185.0–247.0)
Creatinine (mg/dL)	1.02 ± 0.3	1.0 (0.9–1.1)
eGFR (mL/min/1.73 m^2^)	74.3 ± 18.1	75.3 (59.9–88.2)
AST (U/L)	31.7 ± 9.3	31.0 (25.0–35.0)
ALT (U/L)	29.2 ± 8.6	28.0 (24.0–32.0)
INR	1.4 ± 0.5	1.2 (1.1–1.5)
APTT (seconds)	36.8 ± 8.8	34.6 (30.7–41.2)
UA (mg/dL)	39.6 ± 14.1	36.6 (30.5–45.6)
HbA1C (%)	6.04 ± 0.8	5.8 (5.6–6.1)
Fasting glucose (mg/dL)	103.7 ± 19.4	99.0 (92.0–106.0)
TSH (uIU/mL)	2.5 ± 1.3	2.6 (1.5–3.2)
**Comorbidities**		
Paroxysmal AF (%)	131 (73.2)	
Persistent AF (%)	53 (29.6)	
Smoking history (%)	63 (35.2)	
Diagnosed CKD (%)	63 (35.2)	
CAD (%)	58 (32.4)	
AMI history (%)	13 (7.3)	
PCI history (%)	30 (16.8)	
CABG history (%)	2 (1.1)	
PAD (%)	10 (5.6)	
Ischemic stroke history (%)	16 (8.9)	
TIA history (%)	52 (29.05)	
Hemorrhagic stroke history (%)	2 (1.1)	
HF(%)	141 (78.8)	
HFpEF (%)	9 (5.03)	
HFmrEF (%)	14 (7.8)	
HFpEF (%)	119 (66.5)	
NYHA (%)		
I/II	134 (74.9)	
III	2 (1.1)	
IV	0 (0)	
Hypercholesterolemia (%)	84 (46.9)	
Hypothyreoidsm (%)	34 (19.0)	
Hyperthyroidism (%)	11 (6.2)	
**Echocardiography parameters**		
EHRA class:		
1, *n* (%)	2 (1.1)	
2a, *n* (%)	44 (24.6)	
2b, *n* (%)	95 (53.1)	
3, *n* (%)	39 (21.8)	
4, *n* (%)	0 (0)	
LVEF (%)	57.04 ± 8.04	60.0 (55.0–61.0)
LA dimension in LAX (mm)	44.4 ± 8.2	45.0 (43.0–47.0)
LA surface area in Ap4CH (cm^2^)	25.9 ± 3.8	25.6 (23.6–27.9)
LA volume (mL)	118.8 ± 27.4	114.6 (101.7–131.5)
V max LAA before ablation (cm/s)	61.4 ± 22.7	56.0 (46.0–74.0)
LAVI (mL/m^2^)	60.9 ± 14.7	59.1 (50.5–67.9)

Abbreviations: IQR—interquartile range; SD—standard deviation; WBC—white blood cells; HGB—hemoglobin; HCT—Hematocrit; RDW—red blood cell distribution width; PLT—Platelets; ALT—alanine aminotransferase; AST—aspartate aminotransferase; UA—Uric Acid; HbA1c—glycosylated hemoglobin; CKD—Chronic kidney disease; CAD—coronary artery disease; AMI—Acute Myocardial Infarction; PCI—percutaneous coronary intervention; CABG—coronary artery bypass graft; PAD—Peripheral artery disease; TIA—transient ischemic attack; HF—heart failure; HFpEF—Heart Failure with Preserved Ejection Fraction; HFrEF—Heart Failure with reduced Ejection Fraction; HFmrEF—Heart Failure with mildly reduced Ejection Fraction; LA—left atrium; LVEF—Left ventricular ejection fraction; LAX—long axis view; Ap4CH—apical; 4 chamber view; LAVI—left atrial volume index.

**Table 3:** Analyzed MMPs and their examined risk alleles for each metalloproteinase.

Gen		refSNP ID	Record of Changes	Risk Allele
SNP1	MMP1	rs1799750	C − (delC) = 2G	−(absence of C) = 2G
SNP2	MMP2	rs243866	G > A	A
SNP3	MMP2	rs17859821	G > A	Uncertain (often A is protective)
SNP4	MMP2	rs243864	T > G	G
SNP5	MMP3	rs522616	T > C	C
SNP6	MMP9	rs378768	G > A	A
SNP7	MMP9	rs17576	A > G	G
SNP8	MMP12	rs2276109	T > C	T (higher expression)

Abbreviations: MMP—metalloproteinase; refSNP ID—Reference SNP cluster ID; SNP—Single Nucleotide Polymorphism; A, T, C, G—Adenine, Thymine, Cytosine, Guanine.

**Table 4:** Comparison of the frequency of risk alleles in the atrial fibrillation group and in the control group.

Gene (SNP)	Genotype/Allele (refSNP ID)		AF Group	Controls	OR (95%CI)	*p* Values
**MMP1** (**SNP1**)	rs1799750 *		***n* = 173**	***n* = 55**		
	**Genotype**	1G/1G	40 (23.1)	18 (32.7)	1.0 (Ref)	
		1G/2G	77 (44.5)	24 (43.6)	1.4 (0.7–2.7)	0.35
		2G/2G	56 (32.4)	13 (23.6)	1.9 (0.9–4.4)	0.15
	**allele**		*n* = 346	*n* = 110		
		1G	157 (45.4)	60 (54.5)	1.0 (Ref)	
		2G	189 (54.6)	50 (45.5)	1.4 (0.9–2.2)	0.10
**MMP2** (**SNP2**)	**rs243866**		*n* = 179	*n* = 56		
	**Genotype**	G/G	108 (60.3)	32 (57.1)	1.0 (Ref)	
		G/A	62 (34.6)	20 (35.7)	0.9 (0.5–1.7)	0.87
		A/A	9 (5.0)	4 (7.1)	0.7 (0.2–2.3)	0.51
	**allele**		*n* = 358	*n* = 112		
		G	278 (77.6)	84 (75.0)	1.0 (Ref)	
		A	80 (22.3)	28 (25.0)	0.8 (0.5–1.4)	0.61
**MMP2** (**SNP3**)	rs17859821 **		*n* = 178	*n* = 55		
	**genotype**	G/G	132 (74.2)	42 (76.4)	1.0 (Ref)	
		G/A	45 (25.3)	13 (23.6)	1.1 (0.5–2.2)	0.86
		A/A	1 (0.6)	0 (0)	0.96 (0.04–24.1)	1.0
	**allele**		*n* = 356	*n* = 110		
		G	309 (86.8)	97 (88.2)	1.0 (Ref)	
		A	47 (13.2)	13 (11.8)	1.1 (0.6–2.2)	0.87
**MMP2** (**SNP4**)	rs243864 **		*n* = 177	*n* = 56		
	**genotype**	T/T	108 (61.0)	32 (57.1)	1.0 (Ref)	
		T/G	58 (32.8)	18 (32.1)	0.95 (0.5–1.8)	1.0
		G/G	11 (6.2)	6 (10.7)	0.5 (0.2–1.6)	0.37
	**allele**		*n* = 354	*n* = 112		
		T	274 (77.4)	82 (73.2)	1.0 (Ref)	
		G	80 (22.6)	30 (26.8)	0.8 (0.5–1.3)	0.37
**MMP3** (**SNP5**)	**rs522616**		*n* = 179	*n* = 56		
	**genotype**	T/T	130 (72.6)	36 (64.3)	1.0 (Ref)	
		T/C	41 (22.9)	19 (33.9)	0.6 (0.3–1.2)	0.16
		C/C	8 (4.5)	1 (1.8)	2.2 (0.3–18.3)	0.69
	**allele**		*n* = 358	*n* = 112		
		T	301 (84.1)	91 (81.3)	1.0 (Ref)	
		C	57 (15.9)	21 (18.8)	0.8 (0.5–1.4)	0.47
**MMP9?** (**SNP6**)	**rs378768**		*n* = 179	*n* = 56		
	**genotype**	G/G	170 (95.0)	53 (94.6)	1.0 (Ref)	
		G/A	9 (5.0)	3 (5.4)	0.9 (0.2–3.6)	1.0
	**allele**		N = 358	N = 112		
		G	349 (97.5)	109 (97.3)	1.0 (Ref)	
		A	9 (2.5)	3 (2.7)	0.9 (0.2–3.5)	1.0
**MMP9** (**SNP7**)	rs17576 ***		*n* = 178	*n* = 56		
	**genotype**	A/A	70 (39.3)	24 (42.9)	1.0 (Ref)	
		A/G	80 (44.9)	23 (41.1)	1.2 (0.6–2.3)	0.62
		G/G	28 (15.6)	9 (16.1)	1.1 (0.4–2.6)	1.0
	**allele**		*n* = 356	*n* = 112		
		A	220 (61.8)	71 (63.4)	1.0 (Ref)	
		G	136 (38.2)	41 (36.6)	1.1 (0.7–1.7)	0.82
**MMP12** (**SNP8**)	rs2276109 ****		*n* = 178	*n* = 53		
	**genotype**	T/T	121 (68.0)	41 (77.4)	1.0 (Ref)	
		T/C	54 (30.3)	12 (22.6)	1.5 (0.7–3.1)	0.30
		C/C	3 (1.7)	0 (0)	2.4 (0.1–47.3)	0.57
	**allele**		*n* = 356	*n* = 106		
		T	296 (83.1)	94 (88.7)	1.0 (Ref)	
		C	60 (16.9)	12 (11.3)	1.6 (0.8–3.1)	0.22

* lack of detection in 7 patients; ** lack of detection in 2 patients; *** lack of detection in 1 patient; **** lack of detection in 4 patients. Abbreviations: MMP—metalloproteinase; refSNP ID—Reference SNP cluster ID; SNP—Single Nucleotide Polymorphism; A, T, C, G—Adenine, Thymine, Cytosine, Guanine; OR—Odds Ratio.

**Table 5:** Comparison of the frequency of risk alleles in the MMPs studied in patients with recurrence of atrial fibrillation and without recurrence within 3, 6, and 12 months.

Gene (SNP)	Genotype/Allele (refSNP ID)		AF After 3 Months	No AF After 3 Months	OR (95%CI)	AF After 6 Months	No AF After 6 Months	OR (95%CI)	AF After 12 Months	No AF After 12 Months	OR (95%CI)
**MMP1** (**SNP1**)	rs1799750 *		***n* = 86**	***n* = 87**		***n* = 45**	***n* = 128**		***n* = 64**	***n* = 109**	
	**Genotype**	1G/1G	22 (25.6)	18 (20.7)	1.0 (Ref)	12 (26.7)	28 (21.9)	1.0 (Ref)	15 (23.4)	25 (22.9)	1.0 (Ref)
		1G/2G	39 (45.3)	38 (43.7)	0.8 (0.4–1.8)	20 (44.4)	57 (44.5)	0.8 (0.4–1.9)	30 (46.9)	47 (43.1)	1.1 (0.5–2.3)
		2G/2G	25 (29.1)	31 (35.6)	0.6 (0.3–1.5)	13 (28.9)	43 (33.6)	0.7 (0.3–1.8)	19 (29.7)	37 (33.9)	0.9 (0.4–2.0)
	**allele**		*n* = 172	*n* = 174		*n* = 90	*n* = 256		*n* = 128	*n* = 218	
		1G	83 (48.3)	74 (42.5)	1.0 (Ref)	44 (48.9)	113 (44.1)	1.0 (Ref)	60 (46.9)	97 (44.5)	1.0 (Ref)
		2G	89 (51.7)	100 (57.5)	0.8 (0.5–1.2)	46 (51.1)	143 (55.9)	0.8 (0.5–1.3)	68 (53.1)	121 (55.5)	0.9 (0.6–1.4)
**MMP2** (**SNP2**)	**rs243866**		*n* = 90	*n* = 89			*n* = 48	*n* = 131		*n* = 67	*n* = 112
	**Genotype**	G/G	53 (58.9)	55 (61.8)	1.0 (Ref)	25 (52.1)	83 (63.4)	1.0 (Ref)	36 (53.7)	72 (64.3)	1.0 (Ref)
		G/A	33 (36.7)	29 (32.6)	1.2 (0.6–2.2)	19 (39.6)	43 (32.8)	1.5 (0.7–3.0)	27 (40.3)	35 (31.2)	1.5 (0.8–2.9)
		A/A	4 (4.4)	5 (5.6)	0.8 (0.2–3.3)	4 (8.3)	5 (3.8)	2.7 (0.7–10.7)	4 (6.0)	5 (4.5)	1.6 (0.4–6.3)
	**allele**		*n* = 180	*n* = 178		*n* = 96	*n* = 262		*n* = 134	*n* = 224	
		G	139 (77.2)	139 (78.1)	1.0 (Ref)	69 (71.9)	209 (79.8)	1.0 (Ref)	99 (73.9)	179 (79.9)	1.0 (Ref)
		A	41 (22.8)	39 (21.9)	1.1 (0.6–1.7)	27 (28.1)	53 (20.2)	1.5 (0.9–2.6)	35 (26.1)	45 (20.1)	1.4 (0.8–2.3)
**MMP2** (**SNP3**)	rs17859821 **		*n* = 89	*n* = 89			*n* = 47	*n* = 131		*n* = 67	*n* = 111
	**genotype**	G/G	63 (70.8)	69 (77.5)	1.0 (Ref)	30 (63.8)	102 (77.9)	1.0 (Ref)	52 (77.6)	80 (72.1)	1.0 (Ref)
		G/A	25 (28.1)	20 (22.5)	1.4 (0.7–2.7)	16 (34.0)	29 (22.1)	1.9 (0.9–3.9)	14 (20.9)	31 (27.9)	0.7 (0.3–1.4)
		A/A	1 (1.1)	0 (0)	3.3 (0.1–82.1)	1 (2.1)	0 (0)	10.1 (0.4–254.1)	1 (1.5)	0 (0)	4.6 (0.2–115.2)
	**allele**		*n* = 178	*n* = 178		*n* = 94	*n* = 262		*n* = 134	*n* = 222	
		G	151 (84.8)	158 (88.8)	1.0 (Ref)	76 (84.8)	233 (88.9)	1.0 (Ref)	118 (88.1)	191 (86.0)	1.0 (Ref)
		A	27 (15.2)	20 (11.2)	1.4 (0.8–2.6)	18 (15.2)	29 (11.1)	1.9 (1.0–3.6)	16 (11.9)	31 (14.0)	0.8 (0.4–1.6)
**MMP2** (**SNP4**)	rs243864 ***		*n* = 89	*n* = 88			*n* = 47	*n* = 130		*n* = 67	*n* = 110
	**genotype**	T/T	53 (59.6)	55 (62.5)	1.0 (Ref)	26 (55.3)	82 (63.1)	1.0 (Ref)	37 (55.2)	71 (64.5)	1.0 (Ref)
		T/G	31 (34.8)	27 (30.7)	1.2 (0.6–2.3)	17 (36.2)	41 (31.5)	1.3 (0.6–2.7)	26 (38.8)	32 (29.1)	1.6 (0.8–3.0)
		G/G	5 (5.6)	6 (6.8)	0.9 (0.2–3.0)	4 (8.5)	7 (5.4)	1.8 (0.5–6.7)	4 (6.0)	7 (6.4)	1.1 (0.3–4.0)
	**allele**		*n* = 178	*n* = 176		*n* = 94	*n* = 260		*n* = 134	*n* = 220	
		T	137 (77.0)	137 (77.8)	1.0 (Ref)	69 (77.0)	205 (77.8)	1.0 (Ref)	100 (74.6)	174 (79.1)	1.0 (Ref)
		G	41 (23.0)	39 (22.2)	1.1 (0.6–1.7)	25 (23.0)	55 (22.2)	1.4 (0.8–2.3)	34 (25.4)	46 (20.9)	1.3 (0.8–2.1)
**MMP3** (**SNP5**)	**rs522616**		*n* = 90	*n* = 89			*n* = 48	*n* = 131		*n* = 67	*n* = 112
	**genotype**	T/T	63 (70.0)	67 (75.3)	1.0 (Ref)	32 (66.7)	98 (74.8)	1.0 (Ref)	46 (68.7)	84 (75.0)	1.0 (Ref)
		T/C	24 (26.7)	17 (19.1)	1.5 (0.7–3.1)	14 (29.2)	27 (20.6)	1.6 (0.7–3.4)	20 (29.8)	21 (18.7)	1.7 (0.9–3.5)
		C/C	3 (3.3)	5 (5.6)	0.6 (0.1–2.8)	2 (4.2)	6 (4.6)	1.0 (0.2–5.3)	1 (1.5)	7 (6.3)	0.3 (0.03–2.2)
	**allele**		*n* = 180	*n* = 178		*n* = 96	*n* = 262		*n* = 134	*n* = 224	
		T	150 (83.3)	151 (84.8)	1.0 (Ref)	78 (81.3)	223 (85.1)	1.0 (Ref)	112 (83.6)	189 (84.4)	1.0 (Ref)
		C	30 (16.7)	27 (15.2)	1.1 (0.6–2.0)	18 (18.7)	39 (14.9)	1.3 (0.7–2.4)	22 (16.4)	25 (11.2)	1.5 (0.8–2.8)
**MMP9?** (**SNP6**)	**rs378768**		*n* = 90	*n* = 89		*n* = 48	*n* = 131		*n* = 67	*n* = 112	
	**genotype**	G/G	85 (94.4)	85 (95.5)	1.0 (Ref)	47 (97.9)	123 (93.9)	1.0 (Ref)	66 (98.5)	104 (92.9)	1.0 (Ref)
		G/A	5 (5.6)	4 (4.5)	1.3 (0.3–4.8)	1 (2.1)	8 (6.1)	0.3 (0.04–2.7)	1 (1.5)	8 (7.1)	0.2 (0.02–1.6)
	**allele**		*n* = 180	*n* = 178		*n* = 96	*n* = 262		*n* = 134	*n* = 224	
		G	175 (97.2)	174 (97.8)	1.0 (Ref)	95 (97.2)	254 (96.9)	1.0 (Ref)	133 (99.3)	216 (96.4)	1.0 (Ref)
		A	5 (2.8)	4 (2.2)	1.2 (0.3–4.7)	1 (2.8)	8 (3.1)	0.3 (0.04–2.7)	1 (0.7)	8 (3.6)	0.2 (0.02–1.6)
**MMP9** (**SNP7**)	rs17576 **		*n* = 89	*n* = 89			*n* = 47	*n* = 131		*n* = 67	*n* = 111
	**genotype**	A/A	34 (38.2)	36 (40.5)	1.0 (Ref)	17 (36.2)	53 (40.5)	1.0 (Ref)	26 (38.8)	44 (39.6)	1.0 (Ref)
		A/G	41 (46.1)	39 (43.8)	1.1 (0.6–2.1)	21 (44.7)	59 (45.0)	1.1 (0.5–2.3)	29 (43.3)	51 (45.9)	1. 0 (0.5–1.9)
		G/G	14 (15.7)	14 (15.7)	1.1 (0.4—2.5)	9 (19.1)	19 (14.5)	1.5 (0.6–3.9)	12 (17.9)	16 (14.4)	1.3 (0.5–3.1)
	**allele**		*n* = 178	*n* = 178		*n* = 94	*n* = 262		*n* = 134	*n* = 222	
		A	109 (61.2)	111 (62.4)	1.0 (Ref)	55 (58.5)	165 (63.0)	1.0 (Ref)	81 (60.4)	139 (62.6)	1.0 (Ref)
		G	69 (38.8)	67 (37.6)	1.0 (0.7–1.6)	39 (41.5)	97 (37.0)	1.2 (0.7–2.0)	53 (39.6)	83 (37.4)	1.1 (0.7–1.7)
**MMP12** (**SNP8**)	rs2276109 **		*n* = 89	*n* = 89			*n* = 48	*n* = 130		*n* = 66	*n* = 112
	**genotype**	T/T	56 (62.9)	65 (73.0)	1.0 (Ref)	30 (62.5)	91 (70.0)	1.0 (Ref)	40 (62.5)	81 (72.3)	1.0 (Ref)
		T/C	31 (34.8)	23 (25.8)	1.6 (0.8–3.0)	18 (37.5)	36 (27.7)	1.5 (0.7–3.1)	24 (37.5)	30 (26.8)	1.6 (0.8–3.1)
		C/C	2 (2.2)	1 (1.1)	2.3 (0.2–26.3)	0 (0)	3 (2.3)	0.4 (0.02–8.5)	2 (0)	1 (0.9)	4.0 (0.4–46.0)
	**allele**		*n* = 178	*n* = 178		*n* = 96	*n* = 260		*n* = 132	*n* = 224	
		T	143 (80.3)	153 (86.0)	1.0 (Ref)	78 (81.3)	218 (83.8)	1.0 (Ref)	104 (78.8)	192 (85.7)	1.0 (Ref)
		C	35 (19.7)	25 (14.0)	1.5 (0.9–2.6)	18 (18.7)	42 (16.2)	1.2 (0.7–2.2)	28 (21.2)	32 (14.3)	1.6 (0.9–2.8)

* lack of detection in 6 patients; ** lack of detection in 1 patient; *** lack of detection in 2 patients;. Abbreviations: MMP—metalloproteinase; refSNP ID—Reference SNP cluster ID; SNP—Single Nucleotide Polymorphism; A, T, C, G—Adenine, Thymine, Cytosine, Guanine; OR—Odds Ratio.

## Data Availability

The original contributions presented in this study are included in the article/Supplementary Material. Further inquiries can be directed to the corresponding author.

## Supplementary Materials

The following supporting information can be downloaded at https://www.mdpi.com/article/10.3390/ijms27156710/s1. References [26,27,28,29,30,31,32,33,34,35,36,37,38,39,40,41,42,43,44,45,46,47] are cited in Supplementary File.
