# Supplementary material for: The Association Between Matrix Metalloproteinase-1, -2, -3, -9, and -12 Gene Polymorphisms and Atrial Fibrillation
Source: Int J Mol Sci. 2026 Jul 27;27(15):6710. doi: 10.3390/ijms27156710 (PMC13467501; doi:10.3390/ijms27156710)
Supplement: Supplementary file 1 [file ijms-27-06710-s001.zip › ijms-4353507-supplementary.pdf]

Supplementary Materials

Table S1. Individual justification for each SNP.

Gene	rsID	Rationale for SNP selection.
MMP-1 −1607 1G/2G	rs1799750	A promoter insertion/deletion polymorphism creates an additional Ets transcription factor binding site in the 2G allele, resulting in increased transcriptional activity of the MMP-1 gene. It is one of the best-characterized functional variants of MMP-1 and has been associated with altered MMP-1 expression and serum concentrations [26, 27, 28, 29].
MMP-2 −1575 G/A	rs243866	A promoter polymorphism is located within a regulatory region of the MMP-2 gene and has been proposed to affect transcription factor binding and gene expression. It has been investigated in numerous studies evaluating MMP-2 regulation and susceptibility to cardiovascular diseases [30, 31, 32,, 33].
MMP-2 −1059 G/A	rs17859821	A promoter-region SNP has been investigated as part of MMP-2 promoter haplotypes in studies of cardiovascular disease. Although evidence for a direct functional effect on transcription is limited, it may contribute to genetic variability within the MMP-2 regulatory region and has been included in haplotype analyses evaluating disease susceptibility and prognosis [18, 34]
MMP-2, −790 G/T	rs243864	A polymorphism has frequently been analyzed together with other MMP-2 promoter variants as part of functional haplotypes influencing gene expression. Because it is part of the regulatory region of the gene it may contribute to variation in MMP-2 expression and disease susceptibility [34, 35, 36]
MMP-3 −1171 5A/6A	rs3025058	A promoter insertion/deletion polymorphism is a well-established functional variant. The 5A allele exhibits higher transcriptional activity than the 6A allele due to reduced binding of a transcriptional repressor, leading to increased MMP-3 expression [37, 38].
MMP-9 −1562 C/T	rs3918242	A functional promoter SNP affects transcriptional regulation of MMP-9. The T allele is associated with higher promoter activity and increased MMP-9 expression. It is one of the most widely investigated

		MMP polymorphisms and has been linked to circulating MMP-9 levels in several populations [39, 40, 41].
MMP-9	rs3787268	An intronic polymorphism that has been investigated in association studies of stroke, intracerebral hemorrhage, and severity of stroke in the course of atrial fibrillation. Although its functional significance remains unclear, it may serve as a marker of genetic variation within the MMP-9 locus and has been included in haplotype-based analyses of disease susceptibility [42, 43, 44].
MMP-9	rs1056628	SNP located in the 3′ untranslated region (3′UTR) of the MMP-9 gene, and it has been reported to affect post-transcriptional regulation by modifying a miR-491-5p binding site. Functional studies suggest that this variant may influence MMP-9 mRNA expression and potentially MMP-9 protein production [45].
MMP-12 −82 A/G	rs2276109	A promoter polymorphism alters an AP-1 transcription factor binding site and influences MMP-12 transcription. Because MMP-12 plays an important role in inflammatory processes and extracellular matrix degradation, this functional SNP is a biologically plausible candidate for influencing disease-related tissue remodeling [46, 47].

The investigated SNPs were selected based on their reported functional relevance and their location within regulatory regions of MMP genes. Most of these variants are promoter polymorphisms known to influence transcription factor binding and gene expression, while rs1056628 is located in the 3′UTR and may affect post-transcriptional regulation. Previous studies have associated these polymorphisms with altered MMP expression, circulating protein levels, extracellular matrix turnover, and susceptibility to inflammatory and fibrotic processes, contributing also to atrial remodeling. Functional polymorphisms in MMPs genes, have been associated with altered MMPS proteins expression and development of atrial fibrillation in some populations, whereas additional variants, may contribute to interindividual variability in MMPs regulation, although direct evidence linking these polymorphisms to atrial remodeling remains limited. They represent a biologically plausible candidate for influencing extracellular matrix remodeling, despite the absence of direct studies in atrial fibrillation [48, 49].

Table S2. MMP variants analysed and genomic coordinates (GRCh38)

Gene	rsID	Chromosome	Position (GRCh38)	Functional region
MMP1	rs1799750	11	≈102,789,335	Promoter (1G/2G indel, ~-1607 bp from TSS)
MMP2	rs243866	16	≈55,487,580	Promoter (-1575 G/A)
MMP2	rs17859821	16	≈55,488,096	Promoter (-1059 G/A)
MMP2	rs243864	16	≈55,488,365	Promoter (-790 G/T)
MMP3	rs522616	11	≈102,781,649	Promoter/regulatory region
MMP9	rs378768	20	≈45,791,320	Regulatory/intronic (transcript-dependent)
MMP9	rs17576	20	≈45,792,978	Exon (missense E402Q)
MMP12	rs2276109	11	≈102,858,405	Promoter (-82 A/G)

The table lists eight polymorphisms across five MMP genes with GRCh38 coordinates, functional context, and intra-cluster distances for 11q22.3. GRCh38 (Genome Reference Consortium Human Build 38).

Table S3. Intra-cluster distances (GRCh38)

Pairs within 11q22.3 cluster	Distance (kb)
MMP1 rs1799750 ↔ MMP3 rs522616	≈7.7
MMP3 rs522616 ↔ MMP12 rs2276109	≈76.8
MMP1 rs1799750 ↔ MMP12 rs2276109	≈69.1

The table S1 and S2 shows the eight polymorphisms analysed in the MMP genes (MMP1, MMP2, MMP3, MMP9, MMP12), together with their coordinates in the GRCh38 reference (chromosome, nucleotide position), functional location (promoter/exon/intron) and — for markers in the 11q22.3 cluster — the approximate distances between the markers (kb).

Table S4. MMP1 - rs1799750 (SNP1) association with AF occurrence (n=228, adjusted by Age+Sex)

Model	Genotype	Control	AF patients	OR (95% CI)	P-value	AIC	BIC
Codominant	1G/1G	18 (32.7%)	40 (23.1%)	1.00	0.078	165.2	189.2
	1G/2G	24 (43.6%)	77 (44.5%)	2.24 (0.89-5.61)			
	2G/2G	13 (23.6%)	56 (32.4%)	3.30 (1.06-10.25)			
Dominant	1G/1G	18 (32.7%)	40 (23.1%)	1.00	0.032	163.7	184.3
	1G/2G-2G/2G	37 (67.3%)	133 (76.9%)	2.55 (1.08-5.98)			
Recessive	1G/1G-1G/2G	42 (76.4%)	117 (67.6%)	1.00	0.15	166.2	186.8
	2G/2G	13 (23.6%)	56 (32.4%)	2.04 (0.75-5.52)			
Overdominant	1G/1G-2G/2G	31 (56.4%)	96 (55.5%)	1.00	0.46	167.8	188.3
	1G/2G	24 (43.6%)	77 (44.5%)	1.35 (0.60-3.02)			
Log-additive	---	---	---	1.86 (1.06-3.29)	0.028	163.5	184

Abbreviations: AF – atrial fibrillation, MMP metalloproteinase; rdID - Reference SNP cluster ID; SNP - Single Nucleotide Polymorphism; A, T, C, G - Adenine, Thymine, Cytosine, Guanine, OR - Odds Ratio; AIC - Akaike information criterion; BIC - Bayesian information criterion.

Table S5. MMP2 -rs243866 (SNP2) association with AF occurrence (n=235, adjusted by Age+Sex)

Model	Genotype	Control	AF patients	OR (95% CI)	P-value	AIC	BIC
Codominant	G/G	32 (57.1%)	108 (60.3%)	1.00	0.37	173.2	197.5
	G/A	20 (35.7%)	62 (34.6%)	0.63 (0.27-1.45)			
	A/A	4 (7.1%)	9 (5%)	0.38 (0.07-2.19)			
Dominant	G/G	32 (57.1%)	108 (60.3%)	1.00	0.2	171.5	192.3
	G/A-A/A	24 (42.9%)	71 (39.7%)	0.59 (0.26-1.31)			
Recessive	G/G-G/A	52 (92.9%)	170 (95%)	1.00	0.37	172.4	193.2
	A/A	4 (7.1%)	9 (5%)	0.45 (0.08-2.51)			
Overdominant	G/G-A/A	36 (64.3%)	117 (65.4%)	1.00	0.35	172.4	193.1
	G/A	20 (35.7%)	62 (34.6%)	0.68 (0.30-1.54)			
Log-additive	---	---	---	0.62 (0.32-1.20)	0.16	171.3	192

Abbreviations: AF – atrial fibrillation, MMP metalloproteinase; rdID - Reference SNP cluster ID; SNP - Single Nucleotide Polymorphism; A, T, C, G - Adenine, Thymine, Cytosine, Guanine, OR - Odds Ratio; AIC - Akaike information criterion; BIC - Bayesian information criterion.

Table S6. MMP2 - rs17859821 (SNP3) association with AF occurrence (n=233, adjusted by Age+Sex)

Model	Genotype	Control	AF patients	OR (95% CI)	P-value	AIC	BIC
Codominant	G/G	42 (76.4%)	132 (74.2%)	1.00	0.91	174.8	198.9
	G/A	13 (23.6%)	45 (25.3%)	1.20 (0.49-2.92)			
	A/A	0 (0%)	1 (0.6%)	NA (0.00-NA)			
Dominant	G/G	42 (76.4%)	132 (74.2%)	1.00	0.69	172.8	193.5
	G/A-A/A	13 (23.6%)	46 (25.8%)	1.20 (0.49-2.92)			
Recessive	G/G-G/A	55 (100%)	177 (99.4%)	1.00	0.88	172.9	193.6
	A/A	0 (0%)	1 (0.6%)	NA (0.00-NA)			
Overdominant	G/G-A/A	42 (76.4%)	133 (74.7%)	1.00	0.69	172.8	193.5
	G/A	13 (23.6%)	45 (25.3%)	1.20 (0.49-2.92)			
Log-additive	---	---	---	1.20 (0.49-2.92)	0.69	172.8	193.5

Abbreviations: AF – atrial fibrillation, MMP metalloproteinase; rdID - Reference SNP cluster ID; SNP - Single Nucleotide Polymorphism; A, T, C, G - Adenine, Thymine, Cytosine, Guanine, OR - Odds Ratio; AIC - Akaike information criterion; BIC - Bayesian information criterion.

Table S7. MMP2 - rs243864 (SNP4) association with AF occurrence (n=233, adjusted by Age+Sex)

Model	Genotype	Control	AF patients	OR (95% CI)	P-value	AIC	BIC
Codominant	T/T	32 (57.1%)	108 (61%)	1.00	0.085	169.9	194.1
	T/G	18 (32.1%)	58 (32.8%)	0.73 (0.31-1.76)			
	G/G	6 (10.7%)	11 (6.2%)	0.17 (0.04-0.82)			
Dominant	T/T	32 (57.1%)	108 (61%)	1.00	0.17	171	191.7
	T/G-G/G	24 (42.9%)	69 (39%)	0.57 (0.26-1.28)			
Recessive	T/T-T/G	50 (89.3%)	166 (93.8%)	1.00	0.035	168.4	189.1
	G/G	6 (10.7%)	11 (6.2%)	0.19 (0.04-0.88)			
Overdominant	T/T-G/G	38 (67.9%)	119 (67.2%)	1.00	0.75	172.7	193.4
	T/G	18 (32.1%)	58 (32.8%)	0.87 (0.38-2.02)			
Log-additive	---	---	---	0.53 (0.28-1.01)	0.053	169.1	189.8

Abbreviations: AF – atrial fibrillation, MMP metalloproteinase; rdID - Reference SNP cluster ID; SNP - Single Nucleotide Polymorphism; A, T, C, G - Adenine, Thymine, Cytosine, Guanine, OR - Odds Ratio; AIC - Akaike information criterion; BIC - Bayesian information criterion.

Table S8. MMP3 - rs522616 (SNP5) association with AF occurrence (n=235, adjusted by Age+Sex)

Model	Genotype	Control	AF patients	OR (95% CI)	P-value	AIC	BIC
Codominant	T/T	36 (64.3%)	130 (72.6%)	1.00	0.12	171	195.2
	T/C	19 (33.9%)	41 (22.9%)	0.52 (0.22-1.23)			
	C/C	1 (1.8%)	8 (4.5%)	4.51 (0.31-65.26)			
Dominant	T/T	36 (64.3%)	130 (72.6%)	1.00	0.32	172.2	193
	T/C-C/C	20 (35.7%)	49 (27.4%)	0.66 (0.29-1.50)			
Recessive	T/T-T/C	55 (98.2%)	171 (95.5%)	1.00	0.15	171.2	191.9
	C/C	1 (1.8%)	8 (4.5%)	5.44 (0.38-77.10)			
Overdominant	T/T-C/C	37 (66.1%)	138 (77.1%)	1.00	0.099	170.5	191.3
	T/C	19 (33.9%)	41 (22.9%)	0.49 (0.21-1.14)			
Log-additive	---	---	---	0.89 (0.46-1.75)	0.74	173.1	193.9

Abbreviations: AF – atrial fibrillation, MMP metalloproteinase; rdID - Reference SNP cluster ID; SNP - Single Nucleotide Polymorphism; A, T, C, G - Adenine, Thymine, Cytosine, Guanine, OR - Odds Ratio; AIC - Akaike information criterion; BIC - Bayesian information criterion.

Table S9. MMP9 - rs17576 (SNP7) association with AF occurrence (n=234, adjusted by Age+Sex)

Model	Genotype	Control	AF patients	OR (95% CI)	P-value	AIC	BIC
Codominant	A/A	24 (42.9%)	70 (39.3%)	1.00	0.33	173	197.2
	G/A	23 (41.1%)	80 (44.9%)	1.44 (0.60-3.46)			
	G/G	9 (16.1%)	28 (15.7%)	0.62 (0.21-1.87)			
Dominant	A/A	24 (42.9%)	70 (39.3%)	1.00	0.77	173.1	193.8
	G/A-G/G	32 (57.1%)	108 (60.7%)	1.13 (0.51-2.50)			
Recessive	A/A-G/A	47 (83.9%)	150 (84.3%)	1.00	0.21	171.6	192.4
	G/G	9 (16.1%)	28 (15.7%)	0.52 (0.19-1.43)			
Overdominant	A/A-G/G	33 (58.9%)	98 (55.1%)	1.00	0.22	171.7	192.4
	G/A	23 (41.1%)	80 (44.9%)	1.65 (0.74-3.71)			
Log-additive	---	---	---	0.88 (0.51-1.53)	0.65	173	193.7

Abbreviations: AF – atrial fibrillation, MMP metalloproteinase; rdID - Reference SNP cluster ID; SNP - Single Nucleotide Polymorphism; A, T, C, G - Adenine, Thymine, Cytosine, Guanine, OR - Odds Ratio; AIC - Akaike information criterion; BIC - Bayesian information criterion.

Table S10. MMP12 - rs2276109 (SNP8) association with AF occurrence (n=231, adjusted by Age+Sex)

Model	Genotype	Control	AF patients	OR (95% CI)	P-value	AIC	BIC
Codominant	T/T	41 (77.4%)	121 (68%)	1.00	0.55	170.8	194.9
	T/C	12 (22.6%)	54 (30.3%)	1.05 (0.42-2.63)			
	C/C	0 (0%)	3 (1.7%)	NA (0.00-NA)			
Dominant	T/T	41 (77.4%)	121 (68%)	1.00	0.8	170	190.6
	T/C-C/C	12 (22.6%)	57 (32%)	1.13 (0.45-2.81)			
Recessive	T/T-T/C	53 (100%)	175 (98.3%)	1.00	0.28	168.8	189.5
	C/C	0 (0%)	3 (1.7%)	NA (0.00-NA)			
Overdominant	T/T-C/C	41 (77.4%)	124 (69.7%)	1.00	0.98	170	190.7
	T/C	12 (22.6%)	54 (30.3%)	1.01 (0.41-2.53)			
Log-additive	---	---	---	1.22 (0.52-2.86)	0.65	169.8	190.5

Abbreviations: AF – atrial fibrillation, MMP metalloproteinase; rdID - Reference SNP cluster ID; SNP - Single Nucleotide Polymorphism; A, T, C, G - Adenine, Thymine, Cytosine, Guanine, OR - Odds Ratio; AIC - Akaike information criterion; BIC - Bayesian information criterion.

Table S11. Haplotype association with AF occurrence (n=235, adjusted by Age+Sex)

No.	SNP1	SNP2	SNP3	SNP4	SNP5	SNP6	SNP7	SNP8	Freq	OR (95% CI)	P-value
**1**	2G	G	G	T	T	G	G	T	0.166	1.00	---
**2**	2G	G	G	T	T	G	A	T	0.159	0.78 (0.17 - 3.53)	0.74
**3**	2G	A	G	G	T	G	A	T	0.100	0.24 (0.04 - 1.30)	0.1
**4**	1G	G	G	T	T	G	A	T	0.086	0.12 (0.02 - 0.59)	0.0095
**5**	1G	G	G	T	C	G	A	T	0.085	0.33 (0.07 - 1.57)	0.16
**6**	1G	G	G	T	T	G	A	C	0.047	1.60 (0.02 - 141.99)	0.84
**7**	1G	A	G	G	T	G	G	T	0.045	0.03 (0.00 - 0.29)	0.0031
**8**	1G	G	G	T	C	G	G	T	0.041	0.23 (0.03 - 1.95)	0.18
**9**	2G	G	A	T	T	G	A	T	0.027	3.28 (0.03 - 320.23)	0.61
**10**	1G	G	A	T	T	G	A	C	0.026	0.15 (0.01 - 1.89)	0.14
**11**	1G	A	G	G	T	G	G	C	0.021	0.83 (0.00 - 171.25)	0.95
**12**	1G	G	G	T	T	G	G	T	0.021	2.446E+13	>0.0001
**13**	1G	G	G	T	T	G	G	C	0.016	0.22 (0.01 - 5.40)	0.36
**14**	1G	G	A	T	T	G	G	T	0.016	0.16 (0.01 - 1.78)	0.14
**15**	1G	G	A	T	T	G	A	T	0.015	0.21 (0.01 - 8.69)	0.41
**16**	1G	A	G	G	T	G	A	C	0.014	2.111E+193	<0.0001
**17**	1G	A	G	G	T	G	A	T	0.011	0.08 (0.00 - 4.91)	0.23
**18**	2G	A	A	G	T	G	G	T	0.010	1.09 (0.04 - 31.68)	0.96
**rare**	*	*	*	*	*	*	*	*	0.094	0.23 (0.04 - 1.28)	0.096

Abbreviations: AF – atrial fibrillation, MMP metalloproteinase; rdID - Reference SNP cluster ID; SNP - Single Nucleotide Polymorphism; A, T, C, G - Adenine, Thymine, Cytosine, Guanine, OR - Odds Ratio.

Table S12. Best models of SNP-SNP interactions in AF disease.

	Models	Training Bal. Acc	Testing Bal. Acc.	Sign test (p value)	CVC	Chi-squared	mdrOR (95% CI)
AF patients vs. control	SNP1 (MMP1)+SNP8 (MMP12)	0.581	0.472	0.031	7/10	4.66	3.6 (1.1-12.3

	SNP1 (MMP1)+SNP4 (MMP2)+SNP5 (MMP3)	0.596	0.453	0.019	3/10	5.45	2.5 (1.1-5.5)

Table S13. List of TaqMan probes used to study polymorphisms in MMP genes.

rs ID number		Probe	Concentration of the probe	Polymorphism	Reporter1/Reporter2	Gene
1.	Rs1799750	C__34384693_10	40x	C/-	C/delC [VIC/FAM]	MMP1
2.	Rs243866	C__3225942_10	40x	G>A	A/G [VIC/FAM]	MMP2
3.	Rs17859821	C__33696732_10	40x	G>A	A/G [VIC/FAM]	MMP2
4.	Rs243864	C__26734092_10	40x	T>G	G/T [VIC/FAM]	MMP2
5.	Rs522616	C__3047714_10	40x	T>C	C/T [VIC/FAM]	MMP3
6.	Rs378768	C__659086_10	40x	G>A	A/G [VIC/FAM]	MMP9
7.	Rs17576	C__11655953_10	40x	A>G	A/G [VIC/FAM]	MMP9
8.	Rs2276109	C__15880589_10	40x	T>C	C/T [VIC/FAM]	MMP12

A list of the TaqMan probes used in the study to identify specific metalloproteinase genes under investigation. Genomic DNA was isolated from 500 µl of whole blood using the Blood Mini kit (A&A Biotechnology, Poland) (column-based method) in accordance with the manufacturer's instructions supplied with the kit. The concentration and quality of the isolated DNA were determined using a NanoDrop 2000C spectrophotometer (Thermo Scientific, USA). All samples exhibited an absorbance ratio at 260 nm to 280 nm (A260/A280) within the range of 1.8–2.00. Eight commercial TaqMan-type allele-specific probes were used to analyse single-nucleotide polymorphisms in the MMP-1, MMP-2, MMP-3, MMP-9 and MMP-12 genes: rs1799750, rs243866, rs17859821, rs243864, rs3025058, rs3918242, rs3787268 and rs1056628. A real-time PCR reaction was carried out using a StepOne plus instrument (Applied Biosystems, USA) under the following reaction conditions: enzyme activation for 10 minutes at 95°C, followed by 45 PCR cycles (denaturation for 15 seconds at 95°C; primer annealing and DNA strand extension for 1 minute at 60°C).

Table S14. The frequency of risk alleles in the atrial fibrillation group and in the control group after matching.

			Unadjusted cohort			Propensity score matched cohort		
**Gene (SNP)**	**Genotype/allele (refSNP ID)**		**AF group**	**Control**	SMD	AF group	Control	SMD
**MMP1 (SNP1)**	**rs1799750***		**n=173**	**n=55**		n=173	N=151	
	**Genotype**	1G/1G	40 (23.1)	18 (32.7)	0.18	40 (23.1)	26 (17.2)	0.2
		1G/2G	77 (44.5)	24 (43.6)		77 (44.5)	115 (76.2)	
		2G/2G	56 (32.4)	13 (23.6)		56 (32.4)	10 (6.6)	
**MMP2 (SNP2)**	**rs243866**		N=179	N=56		N=179	N=179	
	**Genotype**	G/G	108 (60.3)	32 (57.1)	-0.05	108 (60.3)	35 (19.5)	-0.38
		G/A	62 (34.6)	20 (35.7)		62 (34.6)	139 (77.6)	
		A/A	9 (5.0)	4 (7.1)		9 (5.0)	5 (2.8)	
**MMP2 (SNP3)**	**rs17859821****		N=178	N=55		N=178	N=179	
	**genotype**	G/G	132 (74.2)	42 (76.4)	0.02	132 (74.2)	153 (85.5)	0.11
		G/A	45 (25.3)	13 (23.6)		45 (25.3)	26 (14.5)	
		A/A	1 (0.6)	0 (0)		1 (0.6)	0 (0)	
**MMP2 (SNP4)**	**rs243864****		N=177	N=56		N=177	N=179	
	**genotype**	T/T	108 (61.0)	32 (57.1)	-0.09	108 (61.0)	35 (19.5)	-0.38
		T/G	58 (32.8)	18 (32.1)		58 (32.8)	139 (77.6)	
		G/G	11 (6.2)	6 (10.7)		11 (6.2)	5 (2.8)	
**MMP3 (SNP5)**	**rs522616**		N=179	N=56		N=179	N=179	
	**genotype**	T/T	130 (72.6)	36 (64.3)	-0.06	130 (72.6)	164 (91.6)	0.23
		T/C	41 (22.9)	19 (33.9)		41 (22.9)	14 (7.8)	
		C/C	8 (4.5)	1 (1.8)		8 (4.5)	1 (0.6)	
**MMP9? (SNP6)**	**rs378768**		N=179	N=56		N=179	N=179	
	**genotype**	G/G	170 (95.0)	53 (94.6)	0.0	170 (95.0)	176 (98.3)	0.03
		G/A	9 (5.0)	3 (5.4)		9 (5.0)	3 (1.7)	
**MMP9 (SNP7)**	**rs17576*****		N=178	N=56		N=178	N=179	
	**genotype**	A/A	70 (39.3)	24 (42.9)	0.03	70 (39.3)	118 (65.9)	0.39
		A/G	80 (44.9)	23 (41.1)		80 (44.9)	55 (30.7)	
		G/G	28 (15.6)	9 (16.1)		28 (15.6)	6 (3.3)	
**MMP12 (SNP8)**	**rs2276109******		N=178	N=53		N=178	N=177	
	**genotype**	T/T	121 (68.0)	41 (77.4)	0.11	121 (68.0)	127 (71.7)	0.06
		T/C	54 (30.3)	12 (22.6)		54 (30.3)	50 (28.3)	
		C/C	3 (1.7)	0 (0)		3 (1.7)	0 (0)	

SMD: Standard mean difference.

Table S15. Impact of propensity score matching on logistic regression analysis of risk factors associated with AF.

	Unadjusted cohort			Propensity score matched cohort		
	Wald Stat	OR (95%CI)	p	Wald Stat.	OR (95%CI)	p
**Intercept**	25.4	0.00 (0.00)	<0.0001	44.7	0.00 (0.00)	<0.0001
**Male sex**	24.4	21.04 (6.3-70.4)	<0.0001	41.2	31.9 (11.1-91.7)	<0.0001
**SNP1 (MMP1)**	6.5	2.8 (1.3-6.2)	0.011	22.3	7.6 (3.3-17.5)	<0.0001
**SNP2 (MMP2)**	1.3	7.28 (0.2-223.9)	0.26	2.2	10.2 (0.49-211.7)	0.134
**SNP3 (MMP2)**	0.09	0.85 (0.3-2.4)	0.76	0.8	0.63 (0.24-1.7)	0.358
**SNP4 (MMP2)**	1.8	0.1 (0.004-2.7)	0.17	4.58	0.04 (0.002-0.8)	0.032
**SNP5 (MMP3)**	1.7	1.9 (0.7-5.05)	0.19	14.4	8.4 (2.8-25.1)	<0.0001
**SNP6 (MMP9)**	0.8	0.33 (0.03-3.5)	0.36	1.16	0.36 (0.05-2.3)	0.282
**SNP7 (MMP9)**	0.024	0.95 (0.5-1.8)	0.88	8.04	2.7 (1.4-5.2)	0.005
**SNP8 (MMP12)**	3.447	2.85 (0.9-8.6)	0.06	27.2	16.4 (5.7-46.8)	<0.0001
**Age (years)**	28.579	1.1 (1.07-1.15)	<0.0001	11.8	1.07 (1.02-1.1)	0.001
**BMI (kg/m2)**	4.933	1.16 (1.02-1.33)	0.026	21.2	1.4 (1.2-1.6)	<0.0001

References

26. Silva, A.S.D.; Cavalcanti, M.D.S.M.; Belmont, T.F.M., Ximenes R.A.A, Silva, A.V., Nóbrega, D.N., Souza, R.D.S., Farias, I.C.C., Palmeira do Ó, K., Vasconcelos, L.R.S.; et al. The 1G/1G+1G/2G Genotypes of MMP1 rs1799750 Are Associated with Higher Levels of MMP-1 and Are Both Associated with Lipodystrophy in People Living with HIV on Antiretroviral Therapy. *AIDS Res. Hum. Retrovir.* **2021**, *37*, 399–406.
27. Rutter, J.L.; Mitchell, T.I.; Butticè, G., Meyers, J., Gusella, J.F., Ozelius, L.J., Brinckerhoff, C.E. A single nucleotide polymorphism in the matrix metalloproteinase-1 promoter creates an Ets binding site and augments transcription. *Cancer Res.* **1998**, *58*, 5321–5325.
28. Fujimoto, T.; Parry, S.; Urbanek, M., Sammel, M., Macones, G., Kuivaniemi, H., Romero, R., Strauss, J.F. A single nucleotide polymorphism in the matrix metalloproteinase-1 (MMP-1) promoter influences amnion cell MMP-1 expression and risk for preterm premature rupture of the fetal membranes. *J. Biol. Chem.* **2002**, *277*, 6296–6302.
29. Tower, G.B.; Coon, C.I.; Belguise, K., Chalbos, D., Brinckerhoff, C.E. Fra-1 targets the AP-1 site/2G single nucleotide polymorphism (ETS site) in the MMP-1 promoter. *Eur. J. Biochem.* **2003**, *270*, 4216–4225. https://doi.org/10.1046/j.1432-1033.2003.03821.x. PMID: 14519134.
30. Ritter, A.M.V.; de Faria, A.P.; Barbaro, N.R., Sabbatini, A.R., Batista Corrêa, N., Brunelli, V., Fattori, A., Amorim, R., Modolo, R., Moreno, H. The rs243866/243865 polymorphisms in MMP-2 gene and the relationship with BP control in obese resistant hypertensive subjects. *Gene* **2018**, *646*, 129–135.
31. Elahirad, S.; Elieh Ali Komi, D.; Kiani, A., Mohammadi-Noori, E., Vaisi-Raygani, A., Mozafari, H., Bahrehmand, H., Saidi, M., Toupchi-Khosroshahi, V., Salehi, N. Association of Matrix Metalloproteinase-2 (MMP-2) and MMP-9 Promoter Polymorphisms, Their Serum Levels, and Activities with Coronary Artery Calcification (CAC) in an Iranian Population. *Cardiovasc. Toxicol.* **2022**, *22*, 118–129.
32. Vasků, A.; Goldbergová, M.; Izakovicová Hollá, L., Sisková, L., Groch, L., Beránek, M., Tschöplová, S., Znojil, V., Vácha, J. A haplotype constituted of four MMP-2 promoter polymorphisms (-1575G/A, -1306C/T, -790T/G and -735C/T) is associated with coronary triple-vessel disease. *Matrix Biol.* **2004**, *22*, 585–591.
33. Balistreri, C.R.; Allegra, A.; Crapanzano, F., Pisano, C., Triolo, O.E., Argano, V., Candore, G., Lio, D., Ruvolo, G. Associations of rs3918242 and rs2285053 MMP-9 and MMP-2 polymorphisms with the risk, severity, and short- and long-term complications of degenerative mitral valve diseases: A 4.8-year prospective cohort study. *Cardiovasc. Pathol.* **2016**, *25*, 362–370.
34. Hua, Y.; Song, L.; Wu, N., Xie, G., Lu, X., Fan, X., Meng, X., Gu, D., Yang, Y. Polymorphisms of MMP-2 gene are associated with systolic heart failure prognosis. *Clin. Chim. Acta* **2009**, *404*, 119–123.

35. Morgan, A.R.; Han, D.Y.; Thompson, J.M., Mitchell, E.A., Ferguson, L.R. Analysis of MMP2 promoter polymorphisms in childhood obesity. *BMC Res. Notes* **2011**, *4*, 253.
36. Ye, S.; Eriksson, P.; Hamsten, A.; Kurkinen, M., Humphries, S.E., Henney, A.M. Progression of coronary atherosclerosis is associated with a common genetic variant of the human stromelysin-1 promoter which results in reduced gene expression. *J. Biol. Chem.* **1996**, *271*, 13055–13060.
37. Souslova, V.; Townsend, P.A.; Mann, J., van der Loos, C.M., Motterle, A., D'Acquisto, F., Mann, D.A., Ye, S. Allele-specific regulation of matrix metalloproteinase-3 gene by transcription factor NFkappaB. *PLoS ONE* **2010**, *5*, e9902.
38. Zhang, B.; Ye, S.; Herrmann, S.M., de Maat, M., Evans, A., Arveiler, D., Luc, G., Cambien, F., Hamsten, A., Watkins, H. et al. Functional polymorphism in the regulatory region of gelatinase B gene in relation to severity of coronary atherosclerosis. *Circulation* **1999**, *99*, 1788–1794.
39. Wang, L.; Ma, Y.T.; Xie, X., Yang, Y-N., Fu, Z-Y., Liu, F., Li, X.M., Chen, B.D. Association of MMP-9 gene polymorphisms with acute coronary syndrome in the Uygur population of China. *World J. Emerg. Med.* **2011**, *2*, 104–110.
40. Yadav, S.S.; Mandal, R.K.; Singh, M.K., Verma, A., Dwivedi, P., Sethi, R., Usman, K., Khattri, S. High serum level of matrix metalloproteinase 9 and promoter polymorphism—1562 C:T as a new risk factor for metabolic syndrome. *DNA Cell Biol.* **2014**, *33*, 816–822.
41. Gao, N.; Guo, T.; Luo, H., Tu, G., Niu, F., Yan, M., Xia, Y. Association of the MMP-9 polymorphism and ischemic stroke risk in southern Chinese Han population. *BMC Neurol.* **2019**, *19*, 67.
42. Yi, X.; Sui, G.; Zhou, Q., Wang, C., Lin, J., Chai, Z., Zhou, Ju. Variants in matrix metalloproteinase-9 gene are associated with hemorrhagic transformation in acute ischemic stroke patients with atherothrombosis, small artery disease, and cardioembolic stroke. *Brain Behav.* **2019**, *9*, e01294.
43. Yi, X.; Zhou, Q.; Sui, G., Fan, D., Zhang, Y., Shao, M., Han, Z., Luo, H., Lin, J., Zhou, J. Matrix metalloproteinase-9 gene polymorphisms are associated with ischemic stroke severity and early neurologic deterioration in patients with atrial fibrillation. *Brain Behav.* **2019**, *9*, e01291.
44. Bu, Q.; Zhu, Y.; Chen, Q.Y., Li, H., Pan, Y. A polymorphism in the 3′-untranslated region of the matrix metallopeptidase 9 gene is associated with susceptibility to idiopathic calcium nephrolithiasis in the Chinese population. *J. Int. Med. Res.* **2020**, *48*, 300060520980211.
45. Jormsjö, S.; Ye, S.; Moritz, J., Walter, D.H., Dimmeler, S., Zeiher, A.M., Henney, A., Hamsten, A., Eriksson, P. Allele-specific regulation of matrix metalloproteinase-12 gene activity is associated with coronary artery luminal dimensions in diabetic patients with manifest coronary artery disease. *Circ. Res.* **2000**, *86*, 998–1003.
46. Gai, X.; Lan, X.; Luo, Z., Wang, F., Liang, Y., Zhang, H., Zhang, W., Hou, J., Huang, M. Association of MMP-9 gene polymorphisms with atrial fibrillation in hypertensive heart disease patients. *Clin. Chim. Acta* **2009**, *408*, 105–109.
47. Nakano, Y.; Niida, S.; Dote, K., Takenaka, S., Hirao, H., Miura, F., Ishida, M., Shingu, T., Sueda, T., Yoshizumi, M.; et al. Matrix metalloproteinase-9 contributes to human atrial remodeling during atrial fibrillation. *J. Am. Coll. Cardiol.* **2004**, *43*, 818–825.
